# Oneyear longitudinal study on biomarkers of blood–brain barrier permeability in COVID-19 patients

**DOI:** 10.1038/s41598-024-73321-y

**Published:** 2024-09-30

**Authors:** Johanna Wallensten, Sebastian Havervall, Yvonne Power, Marie Åsberg, Kristian Borg, Anna Nager, Charlotte Thålin, Fariborz Mobarrez

**Affiliations:** 1 Academic Primary Health Care Centre, Region Stockholm, Solnavägen 1E, Box 45436, 104 31 Stockholm, Sweden; 2 Department of Clinical Sciences, Karolinska Institute, Danderyd University Hospital, 18288 Stockholm, Sweden; 3 Department of Medical Sciences, Uppsala University, 75185 Uppsala, Sweden; 4 Division of Family Medicine and Primary Health Care, Department of Neurobiology, Care Sciences and Society, Karolinska Institute, 17177 Stockholm, Sweden

**Keywords:** Biomarkers, Medical research, Molecular medicine, Neurology, Pathogenesis

## Abstract

The pathophysiology behind neurological and cognitive sequelae of COVID-19 may be related to dysfunction of the blood-brain barrier (BBB) and previous research indicate transient neuronal injury and glial activation. The aim of this study was to investigate if COVID-19 is related to increased BBB permeability by analyzing leakage of biomarkers such as astrocyte-derived extracellular vesicles (EVs) and S100B. We also investigated whether levels of these biomarkers correlated with self-reported symptoms that persisted > 2 months. The samples in this 1-year follow-up study came from an ongoing longitudinal study of unvaccinated patients hospitalized for COVID-19 at Danderyd University Hospital, Stockholm, Sweden, between April and June 2020. Blood samples were collected at baseline and 4, 8, and 12 months after hospitalization. Information on self-reported clinical symptoms was collected at follow-up visits. A total of 102 patients were enrolled, and 47 completed all follow-up measurements. Peak levels of both biomarkers were observed at 4 months in the subset of 55 patients who were measured at this timepoint. At 12 months, the biomarkers had returned to baseline levels. The biomarkers were not correlated with any of the long-term self-reported symptoms. COVID-19 is associated with transient increased BBB permeability, shown by elevated levels of astrocyte biomarkers in plasma. However, these levels return to baseline 12 months post-infection and do not correlate with long-term symptoms. Further research is needed to unravel the underlying mechanisms causing long-term symptoms in COVID-19 patients.

**Supplementary Information:**

The online version contains supplementary material available at 10.1038/s41598-024-73321-y.

## Introduction

Sequelae are common after COVID-19. A systematic review found that more than half of hospitalized COVID-19 survivors had at least one persistent post-acute sequela of COVID-19 6 months after infection^1^. Mental health disorder was common and almost one fourth (median 23.8%) reported impaired concentration^1^. Moreover, according to a meta-analysis, one third experience fatigue and one out of five cognitive impairment three months after COVID-19^2^.

The pathophysiology behind neurological and cognitive sequelae of COVID-19 may include blood-brain barrier (BBB) dysfunction^3,4^. SARS-CoV-2 has been detected in human brain tissue^5–7^. It may enter the brain through the olfactory mucosa in the nasal cavity^8^ or through circumventricular organs that lack BBB^9^. Additionally, SARS-CoV-2 may also cross the BBB^10^. A variety of proteins are important for maintaining BBB permeability, and increasing data report that inflammatory mediators, such as cytokines and free radicals, can damage the BBB^11^. Increased levels of cytokines such as interleukin-6 (IL-6) and tumor necrosis factor-α (TNF-α) are related to the cytokine storm seen in patients with COVID-19^12^.

Astrocytes, endothelial cells and pericytes are all part of the BBB^13^. Astrocytes are specialized glial cells that link endothelial blood influx and neurons in the central nervous system (CNS) and are essential for BBB functioning^14–17^. Possible biomarkers of astrocyte activation and/or damage and therefore of increased BBB permeability include glial fibrillary acidic protein (GFAP)^18^, aquaporin 4 (AQP4)^19^, and S100 calcium binding protein B (S100B)^20^.

GFAP is a component of the cytoskeletal filament of astrocytes^18^, and AQP4 is a water channel protein mainly expressed in astrocyte end-feet^21^. GFAP and AQP4 can be measured in plasma/serum as soluble proteins or as proteins expressed on extracellular vesicles (EVs). Previous research has found that the lipid membranes of EVs may protect GFAP from enzymes that could degrade it^22^. Thus, measuring GFAP and AQP4 on EVs may better reflect circulating levels of these proteins.

EVs are cell membrane fragments that are released when cells activate or die and are sometimes referred to as microvesicles or microparticles. EVs are detected by their size (approximately 100–1000 nm) and phenotyped through their expression of proteins, which provides information on their cellular origin. When EVs express GFAP and AQP4, it suggests that they originate from astrocytes, so elevated plasma levels of astrocyte-derived EVs may indicate astrocyte activation and/or apoptosis^23^ and therefore increased BBB permeability.

Previous studies suggest that levels of S100B are correlated with BBB integrity^24^. S100B is a calcium-binding protein expressed in many neural cell types^25^, predominantly in mature perivascular astrocytes^20^. According to clinical guidelines, levels of S100B in serum can be used to guide care for people with traumatic brain injury^26^ and may also be useful as a predictive biomarker of the outcome of such injury^27^. Research has also demonstrated a correlation between increased levels of S100B in serum and both disease severity and inflammatory markers in COVID-19 patients^28^. Recently S100B has been found elevated in serum in patients with severe COVID-19, however, neurological symptoms were not associated to the expression of S100B^29^.

To better understand how COVID-19 affects the BBB, we analyzed two independent biomarkers of astrocytes, astrocyte-derived extracellular vesicles (EVs) and S100B, in plasma samples obtained during the acute infection (baseline), and 4, 8, and 12 months after hospitalization for COVID-19. A secondary aim was to investigate whether levels of these biomarkers correlated with self-reported symptoms that persisted for more than 2 months.

## Materials and methods

### Study design and participants

Blood samples were obtained from the ongoing longitudinal COVID-19 Biomarker and Immunity (COMMUNITY) study^30–32^. 102 patients with COVID-19 admitted to Danderyd University Hospital, Stockholm, Sweden, between April and June 2020 were included in the study. At the time of inclusion there were no available COVID-19 vaccines, and all patients were therefore unvaccinated. COVID-19 diagnosis was confirmed with reverse-transcriptase polymerase chain reaction viral RNA detection. Patients younger than 18 years were excluded. Severity of disease was assessed with respiratory index (RI), which measures need for oxygen: 0 = no need, 1 = need for up to 5 L via nasal cannula, 2 = need for > 5 L via cannula or mask, 3 = need for noninvasive ventilation or high-flow nasal cannula, and 4 = need for invasive respiratory treatment.

All surviving patients were invited to follow-up visits 4, 8, and 12 months after inclusion. At each follow-up visit, blood samples were drawn, and patients were asked about symptoms.

### Healthy controls

Pre-pandemic blood samples from healthy individuals (*n* = 42) were primarily utilized as negative controls for instrument calibration and threshold setting. These samples originated from two separate collections conducted in 2008 (*n* = 18) (2009/614-32) and 2015 (*n* = 24) (2015/1533-31/1). All participants in these studies were healthy, with no history of mental illness, stroke, myocardial infarction, or tumor disease. As a secondary objective, these control samples (age and gender matched) also provided a basis for comparison with patient data (baseline comparison).

### Blood sample collection from patients and controls

Blood samples were drawn through an antecubital vein into a citrated tube within 14 days of hospital admission. Samples were centrifuged for 20 min at room temperature at 2000 g within 3 hours of sampling. Platelet poor plasma (PPP) was then carefully pipetted and stored at -80°C until analysis. A similar protocol was used to collect blood samples from healthy controls. However, due to time and personal constraints during the pandemic, samples taken during the acute phase of the infection (baseline) were centrifuged within 3 hours, whereas samples taken from healthy controls and at patient follow-ups were centrifuged within 1 hour.

### Clinical characteristics

At the 4-, 8-, and 12-month follow-ups, patients were interviewed by a research nurse, according to a standardized questionnaire, and asked about presence of symptoms such as loss of taste, loss of smell, difficulty concentrating, nausea, headache, fatigue, and mental fatigue. No clinical examination was undertaken. Patients who reported that they had at least one symptom for more than 2 months after admission to the hospital were classified as having self-reported long-term symptoms.

### Laboratory analyses

#### Analysis of astrocyte-derived extracellular vesicles

EVs were labelled with two astrocyte-specific antibodies: AQP4 monoclonal antibody (ThermoFisher Scientific, Massachusetts, United States) and polyclonal anti-human GFAP (AH Diagnostics, Stockholm, Sweden). The antibodies were labelled with fluorescent dyes according to the manufacturer’s instruction (Abcam, Cambridge, United Kingdom): AQP4 monoclonal antibody with DyLight 755, and anti-human GFAP with DyLight 550. The final concentration of AQP4 antibody was 5 µg/ml, and the final concentration of GFAP antibody was 5 µg/ml.

#### Flow cytometric analysis of extracellular vesicles

**Fig. 1:**
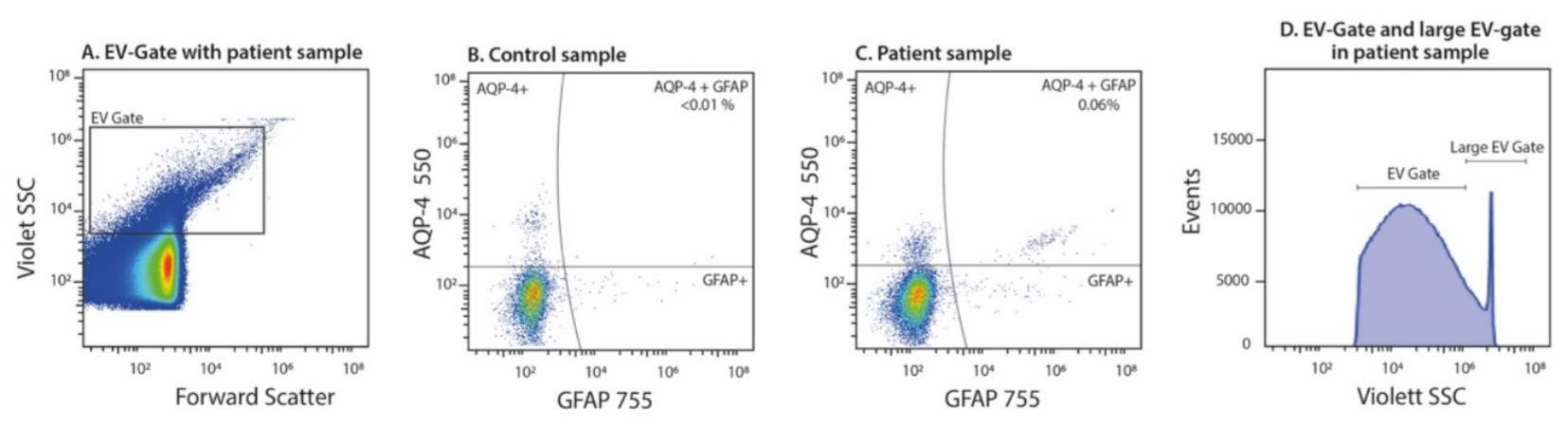
Representative plots of flow cytometric analysis demonstrating the bead and control gates. (A) Dot-plot demonstrating size and complexity for EVs in a patient sample. (B) Sample from a healthy control. Frequency (%) in upper right quadrant (AQP-4 + GFAP) corresponds to number of positive astrocyte-derived EVs events divided by the total number of EV events present in the EV gate. (C) Patient sample from the 4-month follow-up, displaying EVs positive for both AQP4 and GFAP. Frequency (%) in upper right quadrant (AQP-4 + GFAP) corresponds to number of positive astrocyte-derived EVs events divided by the total number of EV events present in the EV gate. (D) Histogram demonstrating large EVs that are outside the normal EV gate (4-month sample).

**Fig. 2:**
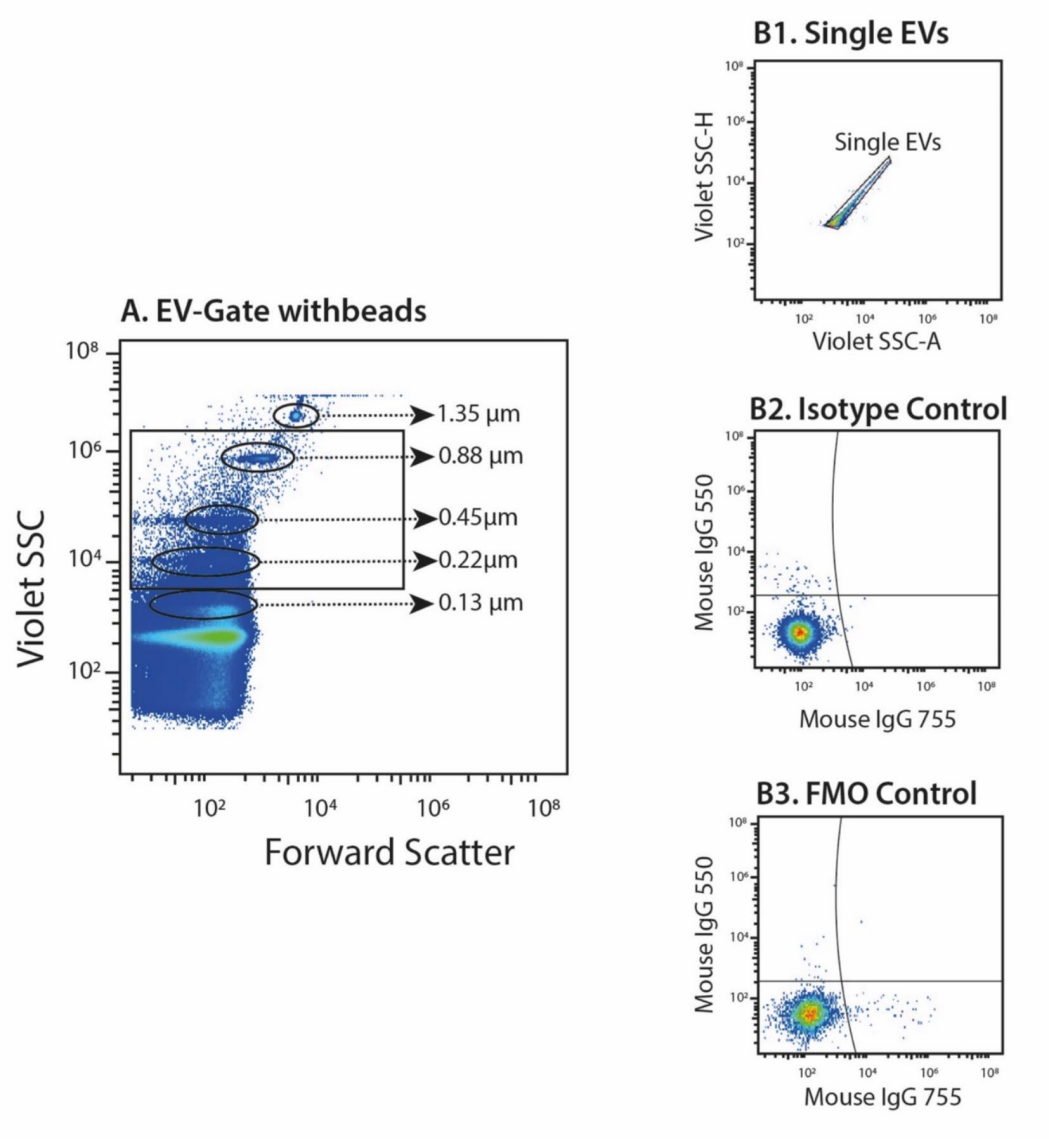
Representative plots of flow cytometric analysis of astrocyte-derived extracellular vesicles (EVs). (A) Dot-plot of Nano fluorescent Yellow Particles used to determine the EVs size range. The beads are gated based on complexity (violet side scatter (SSC)) and forward scatter (size). EVs were defined as vesicles between roughly 0.2 and 1 μm in diameter. Plots demonstrating controls consisting of (B1) gating of single EVs, (B2) Iso-type controls and (B3) fluorescence-minus-one controls.

An EV-enriched pellet was isolated from PPP (500 µl) by high-speed centrifugation as described elsewhere^33^. Briefly, samples were thawed in a water bath and centrifuged initially for 2000*g* for 20 min at room temperature to separate larger debris from plasma. The upper part of the supernatant was then centrifuged at high-speed at 20 800*g* for 45 min at 21°C. The supernatant was discarded and the remaining pellet, which is enriched with EVs, was vortexed. Twenty µl of this EV-enriched pellet was then transferred to a 96 well plate. The plate also contained 5 µl of anti-AQP4 and 5 µl anti-GFAP. The plate was incubated in the dark for 20 min before adding 100 µl of CytoFLEX sheath fluid in order to increase the volume in each well. EVs were then measured using the CytoFLEX flow cytometer (Beckman coulter, Brea, CA, USA) using Violet side scatter as threshold. Unstained EVs, Iso-type controls, single fluorochrome stained EVs, and EVs stained as fluorescence-minus-one (FMO) controls were used to set-up the machine. Samples from healthy controls were used to finetune all the settings and panels. Moreover, samples from the healthy controls and the patients were all run in the same analysis in order to avoid any bias. The EV gate was determined using Spherotech Nano fluorescent Yellow Particles of 0.22 μm, 0.45 μm, 0.88 μm, and 1.35 μm. EVs were defined as vesicles that were between roughly 0.2 and 1 μm and were positive for both AQP4 and GFAP antibodies (Figs. 1 and 2). Results are presented as frequencies of EVs (%): the number of positive astrocyte-derived EVs events divided by the total number of EV events present in the EV gate.

#### Analysis of S100 calcium binding protein B

Plasma concentrations of S100B were quantified (in both control and patient samples) using a commercially available ELISA kit in accordance with the manufacturer’s protocol (Abcam, Cambridge, United Kingdom). The analyses of S100B could not detect levels < 0.011 ng/ml in our samples.

### Statistical methods

All variables were logarithmically transformed, and a one-way repeated measures ANOVA was carried out to determine longitudinal differences with time as the continuous dependent variable. Only patients with samples available from all four measurement points were included in the ANOVA analysis (*n* = 44). In addition, multiple comparison was performed between each time point (Tukey’s multiple comparisons test). Correlation between S100B and EVs were investigated with Pearson’s test. *P*-values < 0.05 were considered statistically significant. All statistical analyses were performed using the JMP statistical discovery from SAS Institute, version 15.2.1 (Cary, NC, United States) and GraphPad Prism v10 (Boston, MA, United States).

### Ethical considerations

The study was approved by the Swedish Ethical Review Authority (2015/1533-31/1, 2020 -1653, 2009/614-2). Informed consent was obtained from all study participants or from their next-of-kin if the potential participant was incapacitated. All research was performed in accordance with relevant guidelines and regulations and in accordance with the Declaration of Helsinki.

## Results

A total of 102 patients were included in the study. Their median age was 59 years, and 63% were male (Table 1). Patients who died (*n* = 6) or did not respond to repeated invitations were excluded from subsequent follow-ups. Thus, study population at 4 months included 55 participants; the 8-month follow-up, 48; and the 12-month follow up, 47. Age and sex did not significantly impact the correlation between biomarkers and symptoms.

**Table 1:** Patient characteristics describing age, sex and BMI together with patients’ self-reported symptoms ≥ 2 months after hospital admission.

	Patients	Controls	*P*
Patient characteristics (*n* = 55)			
Age (median, IQR)	59 [49;68]	58 [54;69]	0.2
Female sex	37%	36%	0.8
BMI (mean ± SD)	28.9 ± 5.6	24.9 ± 2.9	0.0001
RI at baseline (mean ± SD)	0.9 ± 0.9	.	.
Max RI (mean ± SD)	1.2 ± 1.3		
Self-reported long-term symptoms (≥ 2 months after hospital admission) (*n* = 47)			
Loss of taste (% yes)	49%	.	.
Loss of smell (% yes)	45%	.	.
Difficulty concentrating (% yes)	62%	.	.
Nausea (% yes)	10%	.	.
Headache (% yes)	31%	.	.
Fatigue (% yes)	74%	.	.
Mental fatigue (% yes)	55%	.	.

BMI = body mass index, IQR = interquartile range, RI = respiratory index, SD = standard deviation.

**Fig. 3:**
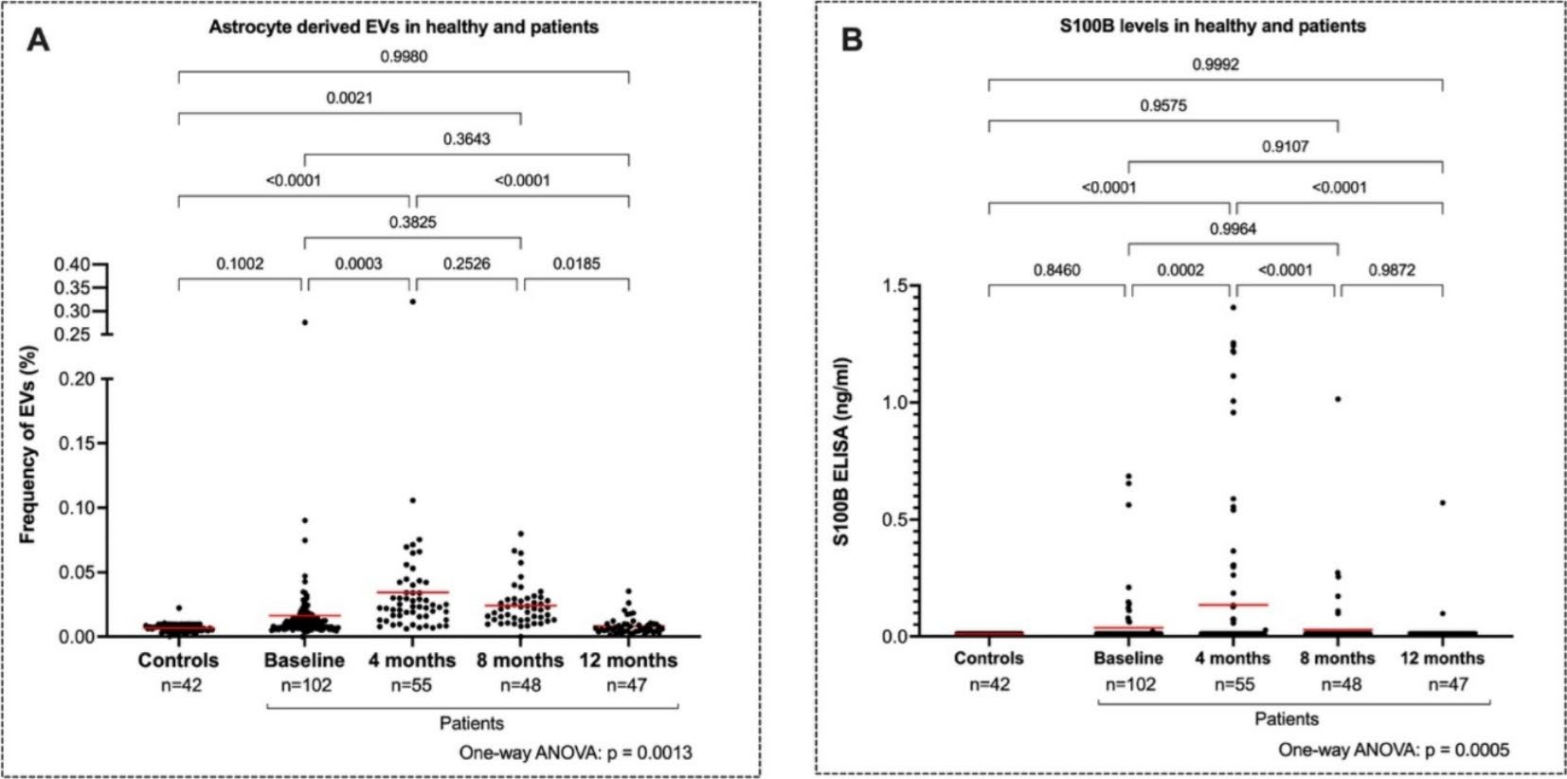
(A) Frequency (%) of astrocyte-derived EVs measured by flow cytometry in controls and patients with Covid-19 at baseline, 4, 8 and 12 months. (B) Plasma levels of S100B (ng/ml) in controls and patients with Covid-19 at the same timepoints. All available data points are shown, though only patients with complete data across all timepoints were included in the statistical analysis. *Tables and their legends*.

Mean levels of astrocyte-derived EVs were two times higher in patients at the 4-month follow-up compared to baseline (*p* < 0.0003) (Fig. 3A). Levels of astrocyte-derived EVs in patients then gradually decreased, and at the 12-month follow-up, were no longer significantly different from those at baseline or compared to control samples (*p* = 0.35 and 1.00 respectively). Paired individual data points is presented as supplementary Fig. 1.

A distinct population of large vesicles outside the EV gate was observed in a subgroup of patients but not in controls (Fig. 1D). The number of patients with these large vesicle populations declined over the study period, from 16 (16%) at baseline, to 9 (16%) at the 4-month follow-up, 4 (8%) at the 8-month follow-up, and 1 (2%) at the 12-month follow-up.

Overall, levels of astrocyte-derived EVs did not correlate with age, sex, BMI, or self-reported clinical symptoms at any time points (Table 2).

The levels of S100B were elevated in patients at the 4-month follow-up compared to the levels in patients at baseline (*p* = 0.0002) similar to the EV analysis (Fig. 3B). Concentrations of S100B in patients then gradually decreased, and at the 12-month follow-up, only two patients had detectable S100B levels. Levels of S100B did not correlate with levels of astrocyte-derived EVs. It is important to note that few patients had S100B levels > 0.011 ng/ml overall during the time points. Healthy controls did not have levels of S100B above the lowest value of quantification (0.011 ng/ml). When the “undetectable” values are excluded from the statistical analysis, the observed changes in S100B levels remain more pronounced yet do not alter the overall interpretation of the data.

**Table 2:** Clinical features correlated to astrocyte-derived EVs and S100B in patients with COVID-19 who came to the four-month follow-up (*n* = 55).

Clinical features	Astrocyte-derived EVs at 4 months (*p*)	S100B at 4 months (*p*)
Age	0.35	0.75
Sex	0.16	0.41
BMI	0.63	0.16
Max RI	0.87	0.76
Loss of taste	0.34	0.18
Loss of Smell	0.35	0.38
Concentration Difficulties	0.24	0.34
Nausea	0.22	0.28
Headache	0.28	0.59
Fatigue	0.24	0.12
Mental Fatigue	0.26	0.38

## Discussion

We measured two independent biomarkers of BBB permeability in peripheral blood, astrocyte-derived EVs and S100B, investigating the potential longitudinal effects of COVID-19 on the BBB. The results showed an increase in both biomarkers following COVID-19, with peak levels four months after infection. These findings suggest that COVID-19 has a direct but transient effect on the BBB.

A range of neurological complications, such as headache, anosmia, and dysgeusia^34–36^ have been associated with COVID-19, and some patients report more chronic symptoms, also known as “post-acute sequelae of COVID-19”, “long COVID”^37^ or as suggested by WHO, “post-COVID condition”^38^. These neurological manifestations could be either a direct consequence of SARS-CoV-2 or an indirect consequence of post-infection complications. One hypothesis is that SARS-CoV-2 activates microglia and astrocytes, inducing an inflammatory response and increasing cytokine production, which results in a feedback loop that affects glia cells in the brain^9^ and subsequently the BBB integrity^3^. Our results, which demonstrate an increase in astrocyte-derived EVs and S100B in peripheral blood after COVID-19, support this hypothesis.

Previous findings of transient neuronal injury and glial activation in patients with COVID-19 are in line with our findings^39,40^. One set of studies investigated plasma levels of neurofilament light chain protein (NfL), a biomarker of neuronal damage^41^, and glial fibrillary acidic protein (GFAP), a biomarker of astrocytic activation or damage^42^. At baseline, NfL was significantly higher in patients with severe disease than in healthy controls, and GFAP was significantly higher in those with moderate and those with severe disease than in healthy controls^39^. After six months, the elevated levels of NfL and GFAP had normalized, regardless of the severity of prior illness or the presence of persisting neurological symptoms^40^. The findings of Kanberg et al. implies that commonly observed neurological consequences following COVID-19 are not due to active neurodegeneration or astroglial activation. Notably, our findings increase the knowledge further by investigating astrocyte-derived EVs in peripheral blood. These findings indicate BBB permeability and, moreover, by investigating a test used in the clinic (S100B), we further investigated the clinical usefulness of monitoring plasma biomarkers to investigate the affection of astrocytes. We observed the same pattern of elevation and normalization but on a different time scale. Astrocyte-derived EVs and S100B were not elevated at baseline in our study. Instead, they were significantly raised four and eight months after infection and decreased to baseline values after 12 months. The differences in findings could be partly explained by differences in patient groups, severity of disease, the timing of the first blood sample, and laboratory methods.

Although levels of both S100B and astrocyte-derived EVs were elevated after COVID-19, we did not find a correlation between these two markers. S100B is mainly found in the cytoplasm of astrocytes^43^ whereas EVs are formed and released from the cell surface. As such, both markers should be seen as independent biomarkers of astrocyte function. Moreover, in an animal model, EVs derived from astrocytes were part of a mechanism for clearing extracellular S100B from the brain^44^, which implies that astrocyte-derived EVs and S100B are two separate markers of astrocyte activation.

Self-reported loss of taste, loss of smell, difficulty concentrating, nausea, headache, fatigue, and mental fatigue were not correlated with elevated levels of brain-derived biomarkers four months after COVID-19. These findings are in line with those of previous research, which found that six months after COVID-19, biomarkers of brain injury had returned to normal levels regardless of neurological symptoms^40^.

During analysis, we detected a group of large vesicles that consistently formed just outside the EV gate. A portion of these large vesicles that were double positive for AQP-4 and GFAP occurred more frequently at baseline and the four-month follow-up than at later follow-ups. One explanation could be the pre-analytical handling EVs (enrichment in a pellet). High-speed centrifugation might cause EVs to compress. Then, because of the light-scatter characteristics of these compressed EVs, the flow cytometer might identify them as larger “vesicles.” However, samples from both patients and controls were centrifuged with the same protocol. If pre-analytical handling were the cause, these large vesicles would occur more randomly across all the samples, including the control samples. Another explanation could be that these larger vesicles are “extracellular vesicles in immune complexes,” or EVs that express various intra-cellular proteins (EV-ICs). It is possible that the cytokine storm that can accompany COVID-19 leads to high levels of EVs from all cells, in particular immune cells. EV-ICs that express various intra-cellular proteins are more antigenic and bind to various antibodies in the patients. This mechanism, including the occurrence of EV-ICs, occurs in people with rheumatic diseases, where EVs have antigenic properties and can bind to autoantibodies of the immunoglobulin G type^45,46^.

### Limitations

There are some limitations in this study. The sample size was small, which means that the findings should be interpreted with caution. It’s important to note that patient and control blood samples were taken at different years. The varying storage times of control samples could potentially influence the results. These samples were measured at three different points in time (2008, 2015, and 2019), independent of this study. While storage time may introduce slight background noise, our analysis showed no significant differences in astrocyte-derived EV levels across these samples, suggesting that storage did not have a meaningful impact on the results.

The primary focus of our study was to investigate the longitudinal changes in COVID-19 patients rather than making direct comparisons between patient and healthy control samples. To establish a baseline, control blood samples were crucial for instrument calibration and threshold setting as they were obtained long before the SARS-CoV-2 outbreak. These controls were processed differently from the patient samples; for instance, control samples were centrifuged within one hour of sampling. On the other hand, due to administrative considerations, patient baseline samples could have remained in the ward for up to three hours prior to centrifugation. Storing samples at room temperature for a prolonged period can result in cell activation and thus increase concentrations of EVs from blood cells in the sample tube^47^. Thus, a direct comparison between controls and patients should be made with caution. However, it’s worth mentioning that the baseline and 12-month levels of astrocyte-derived EVs did not significantly differ from the levels of astrocyte-derived EVs in the control group. Moreover, we detected higher concentrations of EVs and S100B in samples collected at four months, which were centrifuged within the same time span as control samples (one hour). Moreover, our analysis did not account for patients with late-onset symptoms, which is a limitation.

Although GFAP and AQP4 are predominantly expressed by astrocytes, these structures may also be displayed on the surface of other cells that do not reside within the CNS, such as enteric glia cells^48^. However, it is important that all control samples and patients were analyzed simultaneously, and if enteric glia cells had an effect, we would likely observe a different pattern in the results. In addition, astrocytes are the predominant cell type that abundantly express AQP4^49^ and GFAP^50^ and we specifically added two independent assays for detection of astrocyte function/changes (EVs and S100B) to address this limitation. It is important to note that the ELISA assay used in the present study may not be sensitive enough to measure low levels of S100B in plasma. Another limitation is the low frequency of detectable S100B levels, which makes it difficult to determine the significance of these findings. However, even if the levels are low, the highest time points were in accordance with the astrocyte-EV data. In addition, when undetectable S100B values are excluded from the statistical analysis, the observed statistical changes in S100B levels remain (data not shown). It would have been be valuable to include additional cell markers, such as those for endothelial cells and pericytes, to provide a more comprehensive investigation into the integrity of the BBB.

Furthermore, as treatment protocol was not standardized at the time of the study (during pandemic), as such, we have not made adjustments for comorbidities or medication.

A strength of the study is its potential generalizability to other patients hospitalized with SARS-CoV-2 during the study period, when the original virus variant dominated infections in Sweden and before the SARS-CoV-2 vaccines were available, however, we cannot be sure whether the results are also generalizable to vaccinated patients hospitalized with subsequent virus variants.

## Conclusion

Our findings demonstrate a transient increase in astrocyte- derived biomarkers, suggesting that COVID-19 may increase BBB permeability and subsequently astrocyte activation. Self-reported loss of taste, loss of smell, difficulty concentrating, nausea, headache, fatigue, and mental fatigue experienced for longer than 2 months after infection were not correlated with elevated levels of the investigated biomarkers 4 months post-infection. If replicated in larger studies, these results suggest that most symptoms investigated in this study are explained by processes other than the ones indicated by astrocyte-derived EVs and S100B.

## Electronic supplementary material

Below is the link to the electronic supplementary material.

Supplementary Material 1

## Data Availability

The datasets analyzed during the current study is available from the corresponding author on reasonable request.
