# Supplementary material for: Oneyear longitudinal study on biomarkers of blood–brain barrier permeability in COVID-19 patients
Source: Sci Rep. 2024 Sep 30;14:22735. doi: 10.1038/s41598-024-73321-y (PMC11442946; doi:10.1038/s41598-024-73321-y)
Supplement: Supplementary file 1 — Supplementary Material 1 [file 41598_2024_73321_MOESM1_ESM.docx]

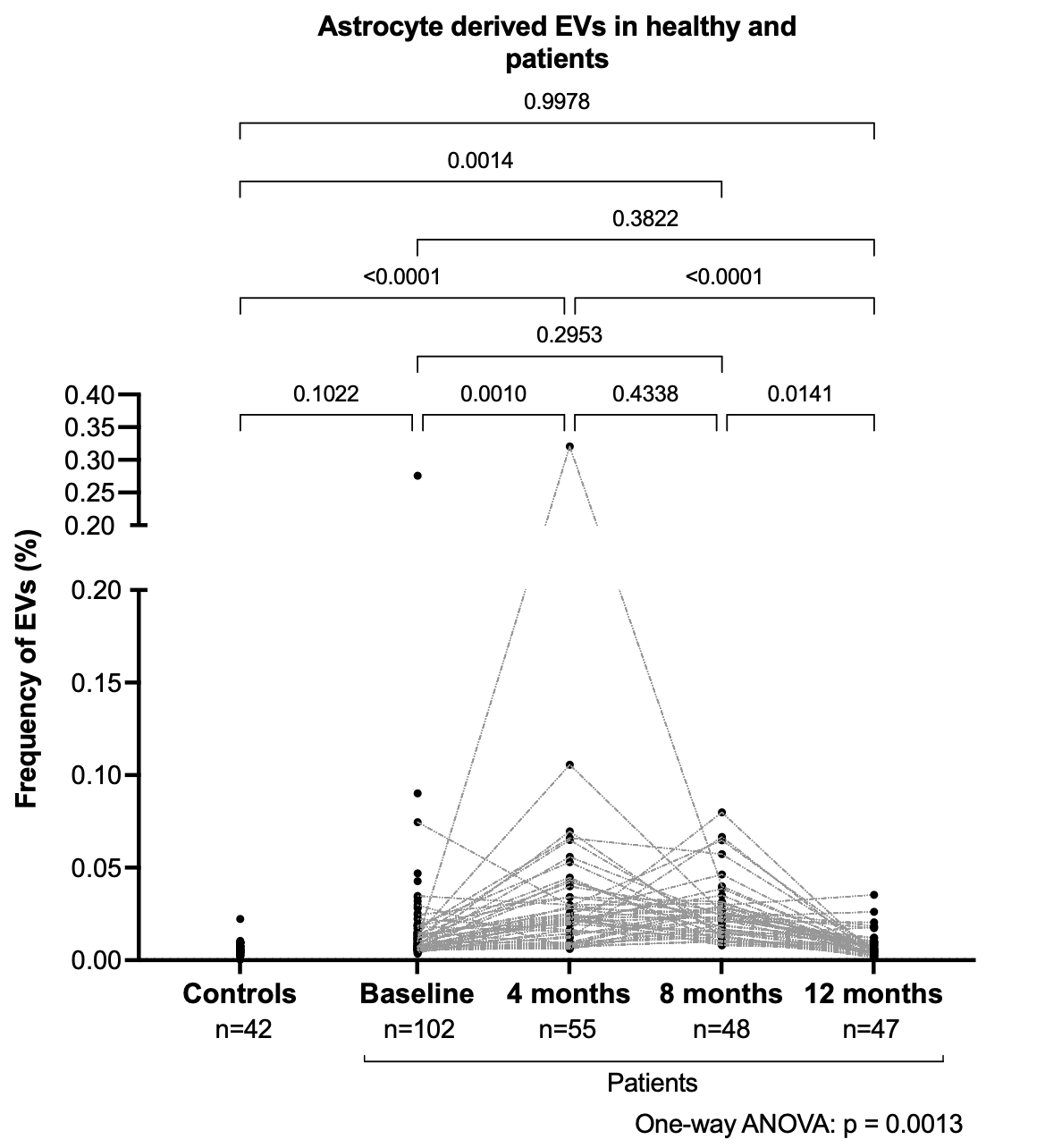


***Supplemental Figure 1****: Paired individual data points for astrocyte-derived EVs in COVID-19 patients at baseline, 4, 8, and 12 months. All available data points are shown, though only patients with complete data across all timepoints were included in the statistical analysis.*
